# Our Healthy Community Conceptual Framework and Intervention Model for Health Promotion and Disease Prevention in Municipalities

**DOI:** 10.3390/ijerph20053901

**Published:** 2023-02-22

**Authors:** Mette Aadahl, Henrik Vardinghus-Nielsen, Paul Bloch, Thea Suldrup Jørgensen, Charlotta Pisinger, Mette Kirstine Tørslev, Charlotte Demant Klinker, Signe Damsbo Birch, Henrik Bøggild, Ulla Toft

**Affiliations:** 1 Center for Clinical Research and Prevention, Bispebjerg and Frederiksberg Hospital, Nordre Fasanvej 57, 2000 Frederiksberg, Denmark; 2 Department of Clinical Medicine, Faculty of Health and Medical Sciences, University of Copenhagen, 2200 N Copenhagen, Denmark; 3 Public Health and Epidemiology Group, Department of Health Science and Technology, Aalborg University, Niels Jernes Vej 14, 9220 Aalborg Ø, Denmark; 4 Steno Diabetes Center Copenhagen, Health Promotion Research, Borgmester Ib Juuls Vej 83, 2730 Herlev, Denmark; 5 Department of Public Health, Faculty of Health and Medical Sciences, University of Copenhagen, 2200 N Copenhagen, Denmark

**Keywords:** community-based intervention, community, partnership, health promotion, co-creation, health in all policies, implementation, supersetting approach, intersectoral action

## Abstract

This paper introduces the conceptual framework and intervention model of Our Healthy Community (OHC), a new, coordinated, and integrated approach towards health promotion and disease prevention in municipalities. The model is inspired by systems-based approaches and employs a supersetting approach for engaging stakeholders across sectors in the development and implementation of interventions to increase health and well-being among citizens. The conceptual model includes a combination of a bottom-up approach emphasizing involvement of citizens and other community-based stakeholders combined with a top-down approach emphasizing political, legal, administrative, and technical support from a variety of councils and departments in local municipality government. The model operates bidirectionally: (1) by pushing political and administrative processes to promote the establishment of conducive structural environments for making healthy choices, and (2) by involving citizens and professional stakeholders at all levels in co-creating processes of shaping their own community and municipality. An operational intervention model was further developed by the OHC project while working with the OHC in two Danish municipalities. The operational intervention model of OHC comprises three main phases and key actions to be implemented at the levels of local government and community: (1) Local government: Situational analysis, dialogue, and political priorities; (2) Community: Thematic co-creation among professional stakeholders; and (3) Target area: Intervention development and implementation. The OHC model will provide municipalities with new tools to improve the citizens’ health and well-being with available resources. Health promotion and disease prevention interventions are developed, implemented, and anchored in the local community by citizens and local stakeholders at municipal and local community levels using collaboration and partnerships as leverage points.

## 1. Introduction

### 1.1. Health Promotion and Prevention of Non-Communicable Diseases

Health care systems face major challenges as life expectancy is increasing, and a growing number of citizens are living with chronic diseases [1,2]. Chronic diseases threaten the health and well-being of the affected individuals and their families and have significant economic consequences for societies by increasing health care costs and reducing productivity. The World Health Organization estimates that up to 80% of chronic diseases could be prevented [2], whereas only 3% of total health care expenses in Europe are allocated to prevention [3].

Evidence shows that health behaviour and chronic diseases are products of a complex interplay between a person’s biological, psychological, environmental, and social circumstances and should therefore be interpreted in a broad socioecological context [4,5]. Promotion of health is central in this broader context. Health promotion is the process of enabling people to increase control over, and to improve their health [6]. As first outlined in the Ottawa charter in 1986, the basic strategies for health promotion are to advocate and boost the factors which encourage health [6]. This requires collaboration across all sectors and enablement of all people to achieve health equity [5,6]. Inequity in health remains an urgent and complex societal challenge, which is largely a result of structural factors disproportionally affecting disadvantaged populations and individuals in our society. Differences in economic, cultural, educational, and social capitals thus influence the capacity of people to exercise healthy living and make proper use of public services [7]. Accordingly, to reduce inequity in health, health promotion and disease prevention must incorporate a variety of strategies and interventions at different levels [7,8]. Research within psychology and behavioural economics shows that healthy choices of individuals are easily influenced by the surrounding environment because much of our behaviour is habitual [9]. Interventions targeting the individual have repeatedly been shown to have mainly short-term effects on health behaviour whereas strategies targeting structural, environmental, and socioeconomic factors are more cost-effective and impactful [10,11]. This calls for new ways to approach inequity in health, including the adoption of a broader health systems approach with a significantly larger emphasis on health promotion and disease prevention [12,13].

In Denmark, municipalities are by law responsible for health promotion and certain aspects of disease prevention [14]. Therefore, Danish municipalities play a key role in developing and implementing preventive health solutions to address the needs of their populations. Most Danish municipalities have approached this responsibility by developing single-stranded, individual-targeted interventions, while struggling with the challenges of establishing intersectoral and interprofessional collaboration mainly used to target structural, environmental, and socioeconomic factors [15]. However, evidence points to the need for coordinated, inter-sectoral, multi-component, and multi-level strategies to promote health and prevent diseases, and the literature highlights the importance of coordination, cooperation, and working together collectively to achieve implementation success [16].

Consequently, there is a need for innovation, including new ways to strengthen intersectoral collaboration, co-creation of solutions with local stakeholders, and optimizing the use of resources in local communities while also focusing on structural conditions for sustainable changes in the health and well-being of citizens. Overall, the complex challenges faced by Danish municipalities call for new approaches and radical system changes to optimise health outcomes for limited public resources [17,18]. 

### 1.2. Our Healthy Community

Our Healthy Community (OHC) develops, implements, and anchors health promotion and disease prevention interventions in the local community by citizens and local stakeholders at municipal and local community levels. Using collaboration and partnerships as leverage points, OHC is a generic model that aims to secure effective and sustainable health promotion interventions across sectors in municipalities and local communities, and to develop and implement approaches and tools that involve and empower citizens to act on their needs and aspirations. 

OHC applies a broad bio-psychosocial concept of health that views health and disease as products of the interplay between a person’s biological, psychological, and social circumstances [4]. This understanding was adopted under the so-called “third revolution of public health” [19]. According to this perspective, health and disease are understood as characteristics that are formed in close interplay between people and the environment in which they live. In accordance with the third revolution of public health, OHC uses the idea of integration of lay knowledge and change in practice that is characterized by: (1) a strong reliance on citizen inputs and participation in decision-making processes regarding public health interventions, (2) an integrated approach that both targets a variety of interrelated risk factors and the social conditions with which they are associated, and (3) deploying activities in a multiplicity of settings. 

OHC is run by academic institutions involved in the development, monitoring, evaluation, and facilitation of intervention processes in cooperation with municipalities. The model will provide municipalities with new tools to improve the citizens’ health and well-being with available resources. This paper introduces the conceptual framework and the intervention model developed by the project. The conceptual framework and the intervention model are based on a theoretical foundation and was refined and adjusted while working with OHC in two Danish municipalities.

## 2. The Conceptual Framework

### 2.1. The Supersetting Approach

The supersetting approach forms the backbone of the conceptual framework of OHC [20] and is rooted in the Ottawa Charter from 1986 [6]. It is a participatory and principles-based intervention strategy, which was developed by the authors and tested in a previous project, the SoL Project that was carried out in selected local communities in Denmark from 2012 to 2015 [21,22]. The SoL Project succeeded in mobilizing unused local resources and in creating measurable changes in health behaviour among citizens [23]. In OHC, we expand the use of the supersetting approach to the municipality level, as previous studies have found that health promotion projects collaborating with local government administrations were more successful in promoting structural changes than projects focusing only on local community engagement [12,20].

According to the supersetting approach, behaviours and health outcomes are results of complex interactions between the knowledge, motivations, and attitudes of citizens, and of the social and physical surroundings of the local community in which they live [24]. This implies that interventions cannot be implemented by using a top-down approach directed by, e.g., city planners, health professionals, or researchers, but demands active engagement and participation of community-based stakeholders and citizens. However, involvement of top-level decision-makers in local government and other partner organisations is mandatory to secure sustainability of structural interventions such as developing new policies and legislation or changing organisations and physical environments [25].

The supersetting approach involves the coordinated engagement of multiple stakeholders in multiple community settings targeting the common overall goal of improving health of local citizens. Interventions based on a supersetting approach are guided by principles to ensure that all actions are not only integrated, but also participatory, empowering, context-sensitive, and knowledge-based. **Integration** refers to the coordination and, where possible, co-implementation of activities sharing features in relation to applied methods, targeted populations, timing, expected outcomes, etc. It also refers to the assimilation of values, approaches, procedures, and standards in established structures and cultures of organizations in the local community and larger society. Finally, integration refers to the cooperation of stakeholders with diverse backgrounds and professions in recognition of the interrelatedness and intersectoral nature of challenges facing society in the 21st century. **Participation** ensures that people are motivated to take ownership of processes of developing and implementing activities. **Empowerment** or **action competence** ensures that people acquire skills and competences to express and act on their visions and aspirations. **Context** ensures that everyday life challenges of citizens and professionals are respected and considered in planning activities. **Knowledge** ensures that scientific knowledge is used to inform action and that scientific knowledge is produced from action.

In accordance with the supersetting approach, OHC builds on the mobilization and use of resources embedded in both the municipality and the local community and on the strengths of social engagement and local ownership as drivers of change processes. By involving multiple stakeholders in multiple settings at municipality and local community levels, activities within individual settings are coordinated and integrated with activities in other settings as a basis for achieving synergistic effects. 

### 2.2. Systems-Based Approaches and Change Models

Systems thinking and selected change models have given further contributions to the conceptual framework of the OHC model. Systems-based approaches have recently been advocated as a means of addressing and understanding complex public health challenges [17,18]. A highly complex public health challenge may be regarded as the outcome of a complex adaptive system where multiple factors interact, and as such it requires a complex intervention to be implemented in a complex system in order to leverage change across the system [26,27,28]. Systems-based approaches require the input and expertise from stakeholders working across various sectors to develop a shared understanding of the complexity of a problem and the surrounding context, and in turn, disrupt the system to change the way it functions [29,30]. 

System changes can be facilitated by different intervention models [31]. These can broadly be divided into democratic and technocratic models [32,33,34]. Democratic models are based on bottom-up strategies whereas technocratic models are based on top-down strategies. Recent evidence shows that the complexity of health promotion and disease prevention necessitates the use of both technocratic and democratic strategies [20,35]. This implies that the health sector of the 21^st^ century requires both (1) structural, environmental, and political change to ensure good and sustainable conditions for making healthy choices and (2) democratic processes involving citizens and local stakeholders in defining, planning, implementing, and evaluating interventions. This will foster local relevance and wide ownership of the intervention within the targeted communities and population groups [36,37,38,39]. 

Many system-change studies highlight the importance of having a solid knowledge base to inform processes of developing interventions that meet the needs and demands of citizens [36,37,38,39]. This knowledge base should preferably include relevant local data on the health condition, well-being, resources, and health behaviours of citizens in the municipality. It should also include the recent scientific evidence and knowledge on the effect of preventive initiatives and health promotion actions [40,41]. Several countries including Iceland, Norway, the Netherlands, and Australia have worked with systematic involvement of local stakeholders and communities in health promotion and disease prevention [41,42,43,44]. “Trøndelagsmodellen” from Norway [42] and “The Icelandic Model” [43] are good examples of operationalization of change models in the public health field. Both models have shown promising results by utilizing data to engage and promote intersectoral action involving multiple stakeholders. In particular, “The Icelandic Model” has successfully managed to combat very high rates of smoking and alcohol consumption among adolescents [43]. The Dutch “LIKE” programme has applied a systems-thinking and participatory action research approach to promote healthy living and healthy weight among teenagers in Amsterdam. This was accomplished by involving stakeholders at family, school, neighbourhood, health care, and city levels [44]. For the purpose of learning how to prevent chronic diseases through complex interventions and working in partnerships, the Australian Prevention Partnership Centre has systematically gathered knowledge on how these intersectoral partnerships between policymakers, practitioners, and researchers are operationalized [41]. Moreover, to promote intersectoral action, the WHO has initiated the Health in All Policies (HiAP) initiative [45]. HiAP approaches include five key elements: (1) promoting health and equity, (2) supporting intersectoral collaboration, (3) creating co-benefits for multiple partners, (4) engaging stakeholders, and (5) creating structural or process change. HiAP is expanding worldwide, and many states have developed implementation guidelines and principles for their local governments [46,47].

## 3. The Conceptual Model

**Figure 1:**
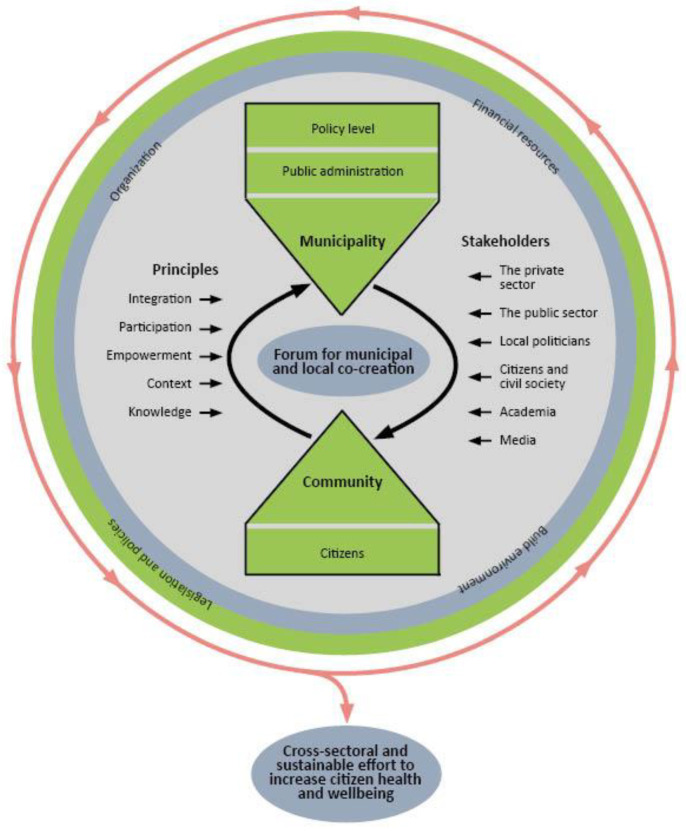
Conceptual framework of the OHC model for prevention and health promotion at municipality level.

The conceptual model of OHC draws on learnings and evidence from the above conceptual framework. The OHC model has three entry points of engagement: society, municipality, and community (Figure 1). The supersetting approach provides guiding principles for developing and implementing interventions together with stakeholders at the levels of local government and community. At the local government level, the supersetting comprises all relevant sectors and departments in the public administration in accordance with the HiAP principles. Actions within individual departments are coordinated and integrated with actions in other departments to promote synergy and coherence. To accommodate for local priorities and contextual conditions at all levels of organisation, the model is flexible and does not prescribe which sectors, departments, organisations, or population groups to involve or which interventions to implement. At the community level, the supersetting comprises all relevant stakeholders and settings in geographical target areas such as towns or neighbourhoods. Actions within individual settings are coordinated and integrated with actions in other settings to promote synergy and coherence. In the interface between the local government and the community, a forum is established for cooperation, innovation, and co-creation of specific activities between stakeholders from bottom and top. Thereby, the conceptual model includes a combination of a bottom-up approach emphasizing involvement of citizens and other community-based stakeholders together with a top-down approach emphasizing political, legal, administrative, and technical support from a variety of councils and departments in local municipality government. The model operates bidirectionally: (1) by pushing political and administrative processes to promote the establishment of conducive structural environments for making healthy choices, and (2) by involving citizens and professional stakeholders at all levels in co-creating processes of shaping their own community and municipality. 

## 4. The Operational Intervention Model

**Figure 2:**
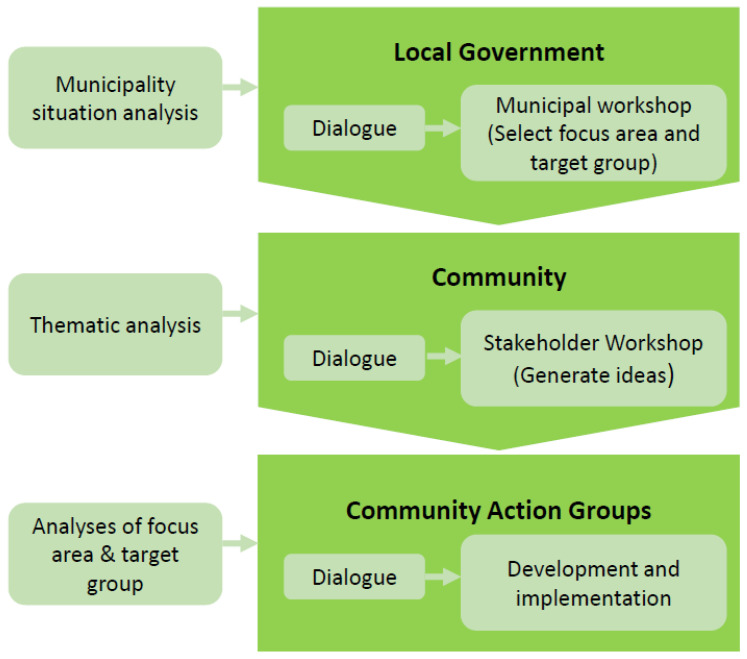
The intervention model of Our Healthy Community.

While the conceptual model above presents the overall conceptual framework of the OHC based on the underlying theoretical foundation, an operational intervention model was further developed by the project while working with the OHC in two Danish municipalities. The operational intervention model of OHC comprises three main phases and key actions to be implemented at the levels of local government and community (Figure 2): (1) Local government: Situational analysis, dialogue, and political priorities; (2) Community: Thematic co-creation among professional stakeholders; and (3) Analysis of focus area and target group: Intervention development and implementation. A local coordinator is employed in the municipality and is dedicated to working with the OHC process.

Phase 1. Local Government: Situational Analysis, Dialogue, and Political Priorities
  - A detailed **municipality situational analysis** of prevailing conditions, resources, and challenges at the municipality level is carried out by project staff. This is undertaken to establish a solid knowledge base for informed decision-making in local government. The analysis includes an extensive analysis of socio-demography, health status, and lifestyle among citizens in the municipality, the organization of health systems and services, as well as existing health promotion and disease prevention initiatives in the municipality. It is mainly based on data from the National Health Profile survey [1]. In Denmark, the National Health Profile survey is carried out as a representative questionnaire-based survey within the adult population in all municipalities every four years (2013, 2017, 2021, etc.). The Municipality Situational Analysis is also based on data and information from other sources, surveys, and projects addressing health conditions, lifestyle, and well-being of citizens at the municipality level, including surveys among children, if available.
  - A series of **dialogue meetings** are held between project staff or project representatives and relevant managers, leaders, and senior staff members in all local municipality administration departments. This is undertaken to present and talk about the project with decision-makers who may or may not consider the core functions of their department to relate to health promotion and disease prevention. The meetings are also held to gain knowledge on the tasks and duties of the departments from the perspective of decision-makers and how this relates to health, if at all. Each dialogue meeting involves a project researcher, the local project coordinator, and 2–5 public officials. It is concluded by an invitation to the department to participate in a subsequent workshop for all high-level decision-makers in the public administration and local government.
  - A **municipal workshop** is organized for all high-level department representatives, directors, and elected council members in local government. The primary objective of this workshop is for the public administration and local government to jointly identify a thematic focus area and a primary target group for subsequent preventive intervention. Secondary objectives are for participants to become familiar with each other, to jointly discuss health as an intersectoral issue, and to establish a conducive environment in the administration for interacting and working together across departments and sectors. The workshop is of 3–4 h duration and is organized and facilitated jointly by project staff and core partners in the public administration. Facilitation is supported by various resources including an extract of data from the municipality situational analysis and a summary of the deliberations from the dialogue meetings. Methodologically, the workshop is inspired by “the search conference” approach [48] and includes group work on processes of co-creation, discussion, negotiation, and consensus building among participants.

Phase 2. Community: Thematic Co-Creation among Professional Stakeholders
  D. A detailed **thematic analysis** of social, structural, organizational, and health-related conditions in the municipality that are directly related to the thematic focus area and primary target group for intervention is carried out by project staff. This is conducted to inform the process of bringing the selected focus areas into action. The analysis includes a mapping of relevant stakeholder organizations, physical structures, settings, and environments in the municipality. It also includes an analysis of health and social data from the municipality regarding the specific health topic that has been prioritized for intervention. Finally, the analysis includes evidence of effective solutions obtained from the scientific literature or from other publications presenting findings from projects and initiatives carried out in Denmark or elsewhere.
  E. A series of **dialogue meetings** are held between project staff, representatives from organizations, institutions, and associations from the public sector, the private sector, and the civil society in the municipality. This is undertaken to present and discuss the project with key community-based stakeholders and to understand their perspectives, priorities, and interests in joining the project and contributing to the development and implementation of project interventions at community level. Each dialogue meeting involves a project researcher, the local project coordinator, and 1–3 stakeholder representatives. It is concluded by an invitation to the stakeholder organization to participate in a subsequent workshop for all relevant community-based stakeholders in the municipality.
  F. A **stakeholder workshop** is organized for representatives from all community-based stakeholder organizations in the municipality who are interested and considered relevant to the thematic focus area and primary target group of the intervention. Eligibility and relevance of stakeholders are determined by core partners in the public administration of the municipality. The primary objective of the workshop is to identify a variety of specific ideas and topics for action within the model of the given thematic focus area and primary target group. Secondary objectives are for participants to get to know each other, to jointly discuss health as an intersectoral issue, and to commence the establishment of a relationship for interacting and working together across organizations and sectors. The workshop is of 3–4 h duration and is organized and facilitated jointly by project staff and core partners in the public administration. Facilitation is supported by various resources including an extract of data from the thematic analysis, a summary of deliberations from the dialogue meetings with stakeholders, and a geographical GIS map of the municipality. Methodologically, the workshop is inspired by “the search conference” approach [36] and includes group work on processes of co-creation, discussion, negotiation, and consensus building among participants.

Phase 3. Target Area: Intervention Development and Implementation
  G. Several **community action groups** are formed by project staff in collaboration with the local coordinator and the municipality based on the outputs of the stakeholder workshop. This is undertaken to establish relevant, intersectoral, and interorganizational partnerships to further develop and test interventions based on their joint priorities and ideas. Prior to this, project staff have reviewed the different ideas for community action that were generated at the stakeholder workshop and aligned them based on proposed topics, settings, target groups, etc. The community action groups thus comprise participants from the stakeholder workshop across public, private, and civic affiliations. New stakeholders may subsequently come onboard while others may leave, depending on the directions taken by the groups. Each community action group strives to develop and implement one or more activities or projects together with relevant citizens and population groups. The community action groups are supported and facilitated by project staff as long as necessary.
  H. A variety of specific **activities or projects** are developed and implemented by the community action groups. For a period of 4–6 months, administrative and technical support is provided to the community action groups by the local project coordinator who organizationally bridges the municipality administration and the project secretariat embedded in an academic partner institution. The project coordinator is partly or fully funded by the project or shared between the project and local government. Project staff mainly provide support to processes of developing and evaluating the activities and projects that are developed by the community action groups. This involves support to the facilitation of development processes and to the evaluation of processes and effects of the intervention. It may also involve support to conduct a contextualised analysis of the selected geographical focus area and target group. To promote synergy and increase impact, the activities and projects that are developed by the different community action groups are coordinated and integrated with each other and with other activities in the municipality. This provides circumstances for developing a coordinated and integrated intervention that is perceived as relevant, has strong local ownership, and is integrated in operations and systems of the municipality.

### Key Assumptions

During preliminary development, testing, and assessment of the OHC model in two Danish municipalities, the project staff identified a variety of factors influencing the collaborative processes and their outcomes. These were subsequently converted into a set of nine key assumptions for a potential successful implementation of the OHC model in a Danish municipality (Table 1). The key assumptions are considered very important for the prospects to implement the OHC model as intended and should be presented to and discussed with decision-makers at the municipality level in early stages of negotiating the terms of engagement and collaboration.

## 5. Discussion

This paper introduces the conceptual framework and intervention model of Our Healthy Community, a new, coordinated, and integrated approach towards health promotion and disease prevention in Danish municipalities. The model is inspired by systems-based approaches and employs a supersetting approach for engaging stakeholders across sectors in the development and implementation of interventions to increase health and well-being among citizens. 

A major novelty of the model is the systematic, stepwise approach and the strong focus on synergy between the local government and the public administration in the municipality on the one hand and the public institutions, private enterprises, non-governmental organisations, and voluntary associations on the other. Previous studies have demonstrated that integrating and collaborating with local government administrations was more efficient than solely focussing on empowering local communities [21,22]. Furthermore, a core criterion of the OHC is that interventions and actions should aim for inclusion of elements of structural change and should be evidence based. To promote sustainable structural changes, it is necessary to also work at the municipality level. We therefore regard the local government and its administration as a central stakeholder that must be included for optimal implementation and sustainability of interventions developed together with community-based stakeholders. The OHC concept includes a strong reliance on citizen input and participation in decision-making regarding the development and implementation of public health interventions. The combination of elements from both technocratic and democratic models of change [33,34] jointly contributes to an intervention strategy with a bottom-up approach emphasizing involvement and co-creation with citizens combined with a top-down approach emphasizing political, administrative, and technical support from the municipality, professional stakeholders, and institutions. Thus, the model works by pushing political and administrative processes to promote the establishment of conducive structural environments for making healthy choices, and by involving citizens and professional stakeholders at all levels in co-creating processes of shaping their own neighbourhood and municipality. 

The OHC model builds on knowledge from system-change studies underlining the importance of a solid knowledge base when developing interventions that meet the needs and demands of citizens [35,36,37,38,39]. “Trøndelagsmodellen” [42] and “The Islandic Model” [43] have successfully demonstrated that utilizing local data and recent scientific evidence in combination with involvement of multiple relevant stakeholders is a promising approach.

Based on our preliminary experience from working with the OHC model in two municipalities, it is evident that the interventions should be developed at different levels to support a synergistic effect of the joint portfolio of interventions in each municipality. Thus, interventions should include structural interventions such as changes in the physical areas of a school or the establishment of a physical meeting place for young people, as was introduced in one local community, together with information campaigns through local media or local organizations, and individual-centred initiatives such as support of children’s participation in local sports clubs and culture associations by strengthening the collaboration between community-based organisations and primary schools. The interventions may be targeting specific population groups or a wider number of the municipality’s citizens. Moreover, in the process of developing interventions, relevant local knowledge should be included as a foundation for the development of targeted and sustainable interventions. This could include local explorative analyses of a specific target group, e.g., young people’s wishes and requests for social networks or the needs, wishes, and resources in a specific local community, area, or institution, e.g., a kindergarten. Finally, implementation of the interventions should take place in close collaboration between the project group, stakeholders in the local action groups involved in developing the intervention, and the local project coordinator. To secure that interventions are anchored and sustained within the municipality, a main focus should be on integrating the interventions into already existing structures in the municipality or the local community such as public departments, community councils, and/or community-based organizations and associations. Furthermore, long-term public policies may facilitate and secure sustainability, whereas short-term political agendas tend to focus more on results at the end of local government election terms and less on sustained long-term health promotion initiatives.

## 6. Conclusions and Perspectives

Our Healthy Community is a new, coordinated, and integrated approach towards health promotion and disease prevention in Danish municipalities. The model applies the supersetting approach for engaging stakeholders across sectors in the development and implementation of interventions to increase health and well-being among citizens. The model is currently being tested in two additional Danish municipalities. Results from the testing, implementation, and evaluation of the OHC model will be reported in subsequent separate publications.

Importantly, for the model to be scalable to municipalities at large, it must be sufficiently flexible to accommodate the contexts and circumstances of each specific municipality at any given point in time. Potential challenges for implementation of the OHC model are sudden and unexpected events such as the COVID-19 crisis or the war in Ukraine necessitating acute and substantial responses from local politicians and municipal administrators, demanding them to adapt to a new and acute agenda of COVID-19 lockdowns or housing refugees from Ukraine. 

## Figures and Tables

**Table 1:** Key assumptions for successful implementation of the OHC model.

Adoption of the OHC model is prioritized and considered a long-term investment in health promotion and disease prevention by the public administration and local government at municipality level.
2. Formalized political approval and commitment by the public administration and local government are required before implementing the OHC model at municipality level.
3. Local government politicians and high-level decision-makers from all sectors and departments at municipality level are invited to jointly select the thematic focus area and primary target group for the intervention.
4. Processes of selecting the thematic focus area and primary target group for the intervention is informed by available evidence and knowledge about health and socioeconomic conditions at municipality level.
5. Activities and projects developed through the OHC model are complementary with existing health promotion and disease prevention initiatives at municipality and community levels.
6. Stakeholders from the public sector, the private sector, and civil society are engaged equitably as co-owners of processes of developing and implementing activities and projects at municipality and community levels.
7. Plans, processes, and developments are widely coordinated and communicated between departments in the public administration and between the public administration and involved community-based stakeholders.
8. Activities and projects developed through the OHC model include relevant strategies, plans, and measures to secure high levels of effectiveness, integration, and sustainability.
9. Lessons learned from implementing the OHC model are documented and used by local government and the public administration to define and support future interventions at municipality level.
